# The DSM-5 - an interview with David Kupfer

**DOI:** 10.1186/1741-7015-11-203

**Published:** 2013-09-12

**Authors:** David Kupfer

**Affiliations:** 1 Department of Psychiatry, University of Pittsburgh School of Medicine, Western Psychiatric Institute and Clinic, 3811 O’Hara St, Pittsburgh, PA 15213, USA

## Abstract

In this podcast we talk to Prof David Kupfer about the challenges, controversies and future directions of DSM-5 by considering other research frameworks and classification systems of disease, and how this revised psychiatric diagnostic manual will impact global mental health classification and the field of medicine.

The podcast for this interview is available at: http://www.biomedcentral.com/sites/2999/download/Kupfer.mp3

## Introduction

**Figure 1:**
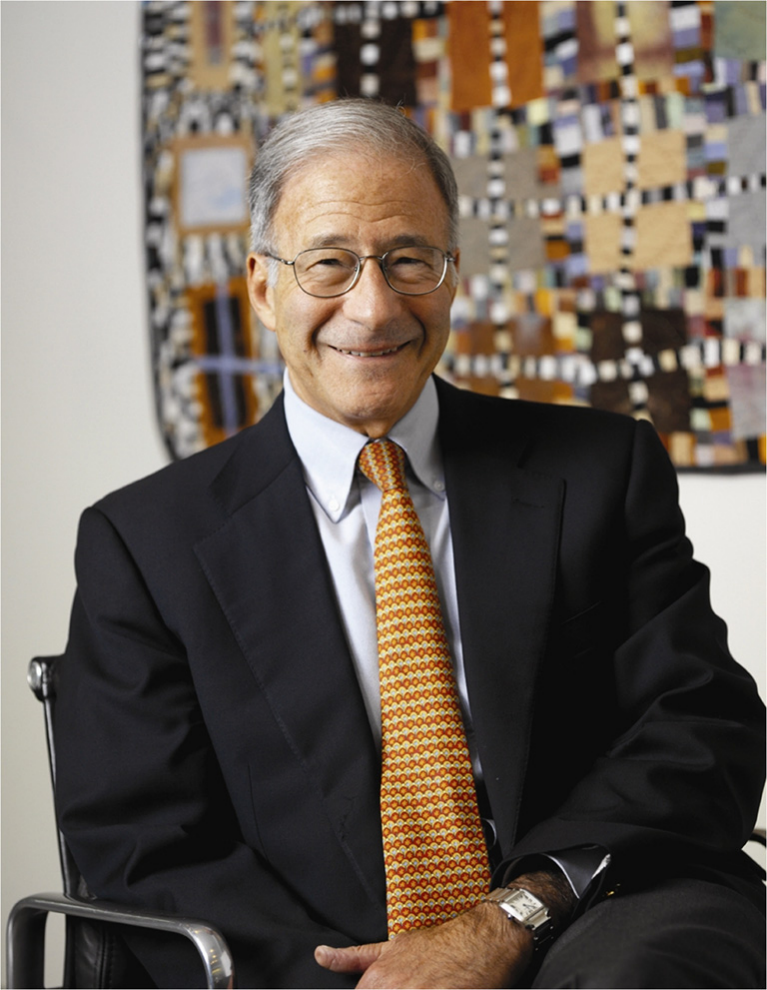
David Kupfer.

Prof David Kupfer chairs the American Psychiatric Association Task Force for the fifth Edition of the Diagnostic and Statistical Manual of Mental Disorders (DSM-5) and is the Thomas Detre Professor of Psychiatry and Professor of Neuroscience and Clinical and Translational Science at the University of Pittsburgh School of Medicine. Prof Kupfer’s research has focused primarily on long-term treatment strategies for recurrent mood disorders, the pathogenesis of depression and the relationship between biomarkers and depression. He is the Founding President of the International Society of Bipolar Disorders, has authored or co-authored a combination of more than 1,021 articles, books and book chapters and has been the recipient of numerous awards and honors (Figure 1).

DSM-5 was released at the American Psychiatric Association’s Annual Meeting in May 2013 and it marked the end of more than a decade’s journey in revising the criteria for the diagnosis and classification of mental disorders.

The podcast for this interview is available at: http://www.biomedcentral.com/sites/2999/download/Kupfer.mp3.

## Edited transcript

### 1. Why was the revision to DSM-5 required?

The revision to DSM-5 was required as it has been nearly 20 years since the Diagnostic and Statistical Manual of Mental Disorders was last updated and we have learned a lot about mental disorders since then. We wanted the fifth edition to help clinicians diagnose mental disorders more precisely. DSM-5 does that by representing the best available science and clinical experience. This new manual is a guidebook that will help clinicians better serve their patients.

### 2. What, in your opinion, are the main challenges for DSM-5?

As with other DSMs, the fifth edition reflects the state of our current scientific knowledge. When we started the process of developing this manual 14 years ago, I think we were all very optimistic that there would be biomarkers and other breakthroughs of a magnitude that would allow us to use biological measures as part of our disorder criteria sets. This has not happened yet, but DSM-5 does provide the best clinical guidebook possible to diagnose mental disorders.

### 3. What are the controversies around DSM-5 in light of other diagnostic frameworks such as the Research Domain Criteria (RDoC)?

DSM and the RDoC programme, which is being developed by the National Institute of Mental Health (NIMH), represent complementary but not competing frameworks. As the RDoC effort takes shape over the next 5 to 10 years, any information or findings stemming from its research agenda will be incorporated into future DSM editions to further strengthen patient care, just as all advances up until now have done. The American Psychiatric Association (APA) and NIMH worked together very closely during the early phases of DSM-5 development and by continuing that partnership we can continue to move the field of mental health forward in meaningful ways.

### 4. Will DSM-5 affect global mental health classification?

We knew that the manual would go far beyond the borders of the United States. Thus we sought to involve as many parts of the world as early as we could. We were able to do this by conducting 13 international conferences between 2003 and 2008. Half of the scientists and clinicians involved in these conferences were from outside North America and represented 39 countries. The conferences were jointly supported by the APA, 3 institutes of the National Institutes of Health [NIH] and the World Health Organisation [WHO]. We also worked closely with WHO to develop harmonization between the diagnostic criteria included in both DSM-5 and the forthcoming 11th Revision of the International Classification of Diseases better known as ICD-11. The DSM and the ICD are companion publications aligned in the technical aspects of diagnostic codes as well as in their organization of disorders. These changes will facilitate improved communication and common use of diagnoses around the globe.

### 5. What are the future directions for DSM-5, for example how will it affect medicine in general?

This is a very important question. First, the American Psychiatric Association is working to make DSM-5 available in multiple formats. In addition to the hardcover, paperback and online versions, they are working on an app design for smart phones and devices and a few other ways to help broaden the methods by which people everywhere can use DSM-5.

In developing the manual itself, we also made the criteria and text more user-friendly for physicians and clinicians outside of psychiatry. This is particularly important because most patients visit their primary care physician and not a psychiatrist when experiencing psychiatric symptoms. DSM-5′s use of definable thresholds across a continuum of normality mirrors that of general medicine’s assessment practice. The well-defined thresholds in DSM-5 will aid physicians in more accurately determining those who need professional help and those who do not.

It’s also important to remember that DSM-5 does not represent an end or the final word. We won’t be waiting another 20 years to update it. Our job in developing the DSM-5 was to synthesize all the advances achieved over the past 20 years and reflect that in the manual while allowing room for growth. This was the most interesting challenge of DSM-5 and also we believe its greatest success. I am personally pleased with the finished product, extremely proud of the contribution that it represents to the future of mental health, and the major contribution of many individuals around the world. We are very eager to see how we can improve it in the coming years.

### 6. Where can I find out more?

See references [1-9].

## Competing interests

DK receives reimbursements, fees, funding or salary from the American Psychiatric Association.

## Pre-publication history

The pre-publication history for this paper can be accessed here:

http://www.biomedcentral.com/1741-7015/11/203/prepub
